# The effectiveness of implementation strategies in improving preconception and antenatal preventive care: a systematic review

**DOI:** 10.1186/s43058-022-00368-1

**Published:** 2022-11-22

**Authors:** Emma Doherty, Melanie Kingsland, John Wiggers, Luke Wolfenden, Alix Hall, Sam McCrabb, Danika Tremain, Jenna Hollis, Milly Licata, Olivia Wynne, Sophie Dilworth, Justine B. Daly, Belinda Tully, Julia Dray, Kylie A. Bailey, Elizabeth J. Elliott, Rebecca K. Hodder

**Affiliations:** 1 Population Health, Hunter New England Local Health District, Locked Bag 10, Wallsend, NSW 2287 Australia; 2 School of Medicine and Public Health, College of Health, Medicine and Wellbeing, The University of Newcastle, Callaghan, NSW Australia; 3 Population Health Research Program, Hunter Medical Research Institute, New Lambton Heights, NSW 2305 Australia; 4 National Centre of Implementation Science, Wallsend, NSW 2287 Australia; 5 School of Psychological Sciences, College of Engineering, Science and Environment, The University of Newcastle, Callaghan, NSW Australia; 6 Faculty of Medicine and Health and Discipline of Child and Adolescent Health, The University of Sydney, Camperdown, NSW 2006 Australia; 7 Sydney Children’s Hospital Network, Kids’ Research Institute, Westmead, NSW 2145 Australia

**Keywords:** Implementation, Antenatal, Preconception, Guidelines, Modifiable risk factors, Systematic review, Meta-analyses

## Abstract

**Background:**

Clinical guideline recommendations for addressing modifiable risk factors are not routinely implemented into preconception and antenatal care. This review assessed the effectiveness of implementation strategies in improving health professional provision of preconception and antenatal care addressing tobacco smoking, weight management and alcohol consumption.

**Methods:**

A systematic review of randomised and non-randomised studies with a parallel comparison group was conducted. Eligible studies used implementation strategy/ies targeted at health professionals to improve at least one element of preconception and/or antenatal care (smoking: ask, advise, assess, assist, arrange; weight/alcohol: assess, advise, refer) compared to usual practice/control or alternative strategies. Eligible studies were identified via CENTRAL, MEDLINE, EMBASE, Maternity and Infant Care, CINAHL and other sources. Random-effects meta-analyses were conducted where appropriate, with other findings summarised using the direction of effect. The certainty of the pooled evidence was assessed using the GRADE approach.

**Results:**

Fourteen studies were included in the review. Thirteen were in the antenatal period and 12 tested multiple implementation strategies (median: three). Meta-analyses of RCTs found that implementation strategies compared to usual practice/control probably increase asking (OR: 2.52; 95% CI: 1.13, 5.59; 3 studies; moderate-certainty evidence) and advising (OR: 4.32; 95% CI: 3.06, 6.11; 4 studies; moderate-certainty evidence) about smoking and assessing weight gain (OR: 57.56; 95% CI: 41.78, 79.29; 2 studies; moderate-certainty evidence), and may increase assessing (OR: 2.55; 95% CI: 0.24, 27.06; 2 studies; low-certainty evidence), assisting (OR: 6.34; 95% CI: 1.51, 26.63; 3 studies; low-certainty evidence) and arranging support (OR: 3.55; 95% CI: 0.50, 25.34; 2 studies; low-certainty evidence) for smoking. The true effect of implementation strategies in increasing advice about weight gain (OR: 3.37; 95% CI: 2.34, 4.84; 2 non-randomised studies; very low-certainty evidence) and alcohol consumption (OR: 10.36; 95% CI: 2.37, 41.20; 2 non-randomised studies; very low-certainty evidence) is uncertain due to the quality of evidence to date.

**Conclusions:**

Review findings provide some evidence to support the effectiveness of implementation strategies in improving health professional delivery of antenatal care addressing smoking and weight management. Rigorous research is needed to build certainty in the evidence for improving alcohol and weight gain advice, and in preconception care.

**Trial registration:**

PROSPERO-CRD42019131691.

**Supplementary Information:**

The online version contains supplementary material available at 10.1186/s43058-022-00368-1.

Contributions to the literature

- This review is the first to examine the effect of implementation strategies in improving health professional provision of preconception and antenatal care addressing priority modifiable risk factors.
- Findings support the use of multiple implementation strategies, including educational materials, educational meetings and reminders, to increase asking and advising about tobacco smoking and assessing gestational weight gain for pregnant women.
- This review highlighted a number of gaps in the literature base, including implementation strategies to improve health professional provision of preconception care and referrals for weight and alcohol consumption in the antenatal period, the cost/cost-effectiveness of implementation strategies and unintentional adverse consequences.

## Background

Maternal tobacco smoking, gestational weight gain outside of recommended ranges and alcohol consumption increase the risk of obstetric complications [1–5] and can lead to adverse health and development outcomes for the child [6]. Clustering of these modifiable risk factors during pregnancy is common [7–9], which further increases the risk and severity of such outcomes [10, 11]. Many countries have adopted guidelines that recommend women who are pregnant or planning a pregnancy should not smoke tobacco or consume alcohol [12]. It is further recommended women eat a healthy diet, be physically active and remain within recommended weight gain ranges during pregnancy [13]. Despite these recommendations, internationally it is estimated that during pregnancy 10% of women smoke [14–16], 10% consume alcohol [17] and 68% gain weight outside of recommended ranges [1, 18, 19].

Systematic review evidence supports the effectiveness of health professional delivered psychosocial interventions in reducing smoking and alcohol consumption during pregnancy [20, 21] and behavioural interventions in preventing excessive weight gain [22, 23]. Routine preconception care may also be effective in modifying these risk factors prior to conception [19, 24]. Consistent with such evidence, clinical guidelines [13, 25–28] recommend that all women receive preventive preconception and antenatal care addressing smoking, weight management (inclusive of nutrition and physical activity) and alcohol consumption. The recommended model for addressing smoking is based on the 5A’s behavioural counselling: ask, advise, assess, assist and arrange [29]. The 5As is informed by the transtheoretical model of behaviour change and was developed by the US Department of Health and Human Services as an evidence-based and practical framework to guide clinician provision of smoking cessation counselling [29]. The 5As has since been adapted for other modifiable risk factors, and this adapted version is recommended by preconception and antenatal clinical guidelines for addressing weight management and alcohol consumption: assess, advise and refer [13, 25–28].

Despite the existence of guideline recommendations, the provision of preconception and antenatal care addressing these risk factors is sub-optimal [30–33]. For example, a study of 1173 pregnant women in the UK found that 13% received preconception advice from a health professional on smoking and alcohol consumption and 10% on recommended weight gain [30]. Similarly, studies in Australia examining antenatal care provision have reported that only 20% of general practitioners routinely address smoking with pregnant women [31] and less than half provide advice on healthy eating (42%) [33], physical activity (39%) [33] and alcohol consumption (32%) [32]. Without routine implementation, the intended benefits of the guidelines in supporting optimal pregnancies and a healthy start to children’s lives will not be fully realised.

Implementation strategies are methods or techniques used to enhance the adoption, implementation and sustainability of evidence-based practices [34]. Cochrane Effective Practice and Organisation of Care (EPOC) developed a taxonomy to classify and organise implementation strategies that are targeted at health professionals, including educational meetings, audit and feedback and reminders (see Table 1 for EPOC taxonomy) [35]. Systematic reviews have shown that such strategies typically improve recommended care practices by 5% to 20% [36–42]. Strategies that are developed using theory and that are tailored to address determinants of practice (e.g. context-specific barriers to implementation as reported by those responsible for delivering care) may yield larger improvements in the range of 9% to 47% [43–45].

Two previous reviews have examined the effectiveness of implementation strategies in supporting health professionals to provide antenatal care addressing a modifiable risk factor [46, 47]. The first, a 2013 review of strategies to support weight management care identified no eligible studies [48]. In the second review, conducted in 2018, meta-analyses of controlled and non-controlled studies showed that implementation strategies significantly increased the provision of smoking care to pregnant women, including asking (Cohen’s d: 0.47; 95% CI: 0.13, 0.81), advising (Cohen’s d: 0.46; 95% CI: 0.02, 0.90) and assisting with quitting (Cohen’s d: 0.65; 95% CI: 0.46, 0.83) [47]. Subgroup analyses found that the use of certain intervention components may have had an impact on the pooled effect, such as theoretical/tailored basis to strategy development, a systems-based strategy, educational outreach visits and audit and feedback for asking about smoking [47]. The review however pooled results from studies comparing implementation strategies to usual practice/control with those comparing alternative strategies, with the latter potentially contributing to an underestimation of effect size. Such pooling also prohibited examination of the comparative effectiveness of different types and combinations of implementation strategies. Since these reviews were published, new studies assessing the effectiveness of implementation strategies to improve antenatal care related to smoking and weight gain have been published but not synthesised [49–53]. Furthermore, no reviews to date have examined the effectiveness of implementation strategies in improving antenatal care addressing alcohol consumption or preconception care addressing any of the three modifiable risk factors.

### Objective

The aim of this systematic review was to examine the effectiveness of implementation strategies in improving health professional provision of preconception and/or antenatal care elements addressing three modifiable risk factors: tobacco smoking, weight management (inclusive of care to improve nutrition and/or physical activity) and/or alcohol consumption.

## Methods

The review was prospectively registered with the International Prospective Register of Systematic Reviews (PROSPERO: CRD42019131691), conducted according to the Cochrane Handbook for Systematic Reviews of Interventions methods [54], and reported in accordance with the Preferred Reporting Items for Systematic Reviews and Meta-Analysis (PRISMA) (Supplementary File 1) [55]. Additional information on review methods is available in the published protocol [56].

### Eligibility criteria

#### Study design

Studies were eligible for inclusion if they were randomised (RCTs) or non-randomised controlled trials with a parallel comparison group. Included studies were restricted to those published in English, or where an English translation was available. There were no eligibility criteria based on the year of study publication, country of origin or length of follow-up.

#### Participants

Studies conducted in any health service (e.g. primary care or hospital clinics) and involved any health professionals (e.g. general practitioners or midwives) who are usual providers of preconception and/or antenatal care were eligible for inclusion.

#### Interventions (implementation strategies)

Studies that aimed to improve preconception and/or antenatal care for the modifiable risk factors of tobacco smoking, weight management or alcohol consumption using one or more of the implementation strategies targeted at healthcare professionals as defined by the EPOC Taxonomy [35] (see Table 1) were eligible.

#### Comparisons

Studies were eligible if they (1) compared the effectiveness of an implementation strategy to improve preconception and/or antenatal care addressing modifiable risk factors with usual practice or control or (2) compared alternative implementation strategies to improve such care.

#### Outcomes

##### Primary outcomes — provision of recommended care

Studies were eligible for inclusion if they reported any quantitative measure of the effectiveness of implementation strategies in improving at least one element of preconception and/or antenatal care for at least one of the eligible modifiable risk factors. Preconception was defined as care to women of childbearing age with the explicit aim of improving health for a future pregnancy. Antenatal was defined as care to women who were currently pregnant. In line with guideline recommendations [13, 25–28], eligible care elements and therefore the primary outcomes of this review, were preconception and/or antenatal care for (i) tobacco smoking: ask (identify smoking status), advise (urge smokers to quit and explain risks), assess (willingness to quit), assist (set a quit date, offer/provide nicotine replacement therapy, referral or other supports) and arrange (follow-up) [57]; (ii) weight management: assess (identify weight gain against recommendations), advise (weight gain, nutrition and physical activity recommendations) and refer (offer support services); and (iii) alcohol consumption: assess (identify alcohol consumption), advise (advise no alcohol and explain risks) and refer (offer support services). The 5A’s behavioural counselling model for addressing tobacco smoking [29], and the adapted version for weight management and alcohol consumption, were chosen for classification of the review’s primary outcomes as they align with preconception and antenatal clinical guideline recommendations [13, 25–28] and are reflected in the literature base reporting on improvements in care provision for these modifiable risk factors. Nutrition and physical activity care elements were considered eligible outcomes in studies targeting weight management care, whereas other nutrition and physical activity care outcomes (e.g. folate advice) were excluded.

Outcome data could include women or health professional self-report surveys, direct observations, medical record audits or other methods. If studies only included one method of data collection (self-report surveys, direct observations or medical record audit), prioritisation of outcome data by data collection method was not required. However, as a number of studies using self-report surveys included data from both women and health professionals, we prioritised inclusion of women’s self-report as it is considered the more reliable of the two when measuring care practices [58].

##### Secondary outcomes

The following secondary outcomes of included studies were also synthesised:

1. Women’s modifiable risk factors: smoking, gestational weight gain, nutrition, physical activity and/or alcohol consumption prior to, or during, pregnancy.
2. Absolute costs or the cost-effectiveness of implementation strategy/ies.
3. Unintentional adverse consequence of implementation strategy/ies.

### Search methods

A search strategy was developed in consultation with a research librarian based on search filters published in previous Cochrane implementation reviews [59, 60] (Supplementary File 2). The search strategy was modified as required and executed across the following electronic databases on the 22^nd^ October 2021: Cochrane Central Register of Controlled Trials, MEDLINE, EMBASE, Maternity and Infant Care, CINAHL, ProQuest Dissertations and Theses, and the World Health Organization International Clinical Trials Registry Platform. Other sources searched were: articles published in the last 5 years in Implementation Science, Journal of Translational Behavioural Medicine, BMC Pregnancy and Childbirth and Midwifery (November 2016 to October 2021), the first 200 results from Google Scholar, and the reference lists of all included studies. The searched journals were chosen through consultation with experts in the field. They were deemed most relevant to the fields of implementation science and translational behavioural sciences and had published a large number of articles relating to the clinical setting/participants of interest for the review. Google Scholar was limited to 200 results as it was a secondary source for the review. A pilot of the strategy had deemed articles beyond this point as very-low relevancy to the review.

### Data collection and analysis

#### Study selection

Following the removal of duplicates, two review authors (ED and one of DT, SM, ML, OW, JD, KB, and BT) independently screened the titles and abstracts of all identified records against the eligibility criteria. Two review authors (ED and one of SM, ML, and OW) independently screened the full texts of potentially eligible studies. Eligibility for study selection was assessed using a standardised pre-piloted screening tool and managed through Covidence. Review authors were not blind to author or journal information [54]. Any discrepancies in screening were resolved by consensus, or with a third reviewer.

#### Data extraction

Two review authors (ED and one of SM and ML) independently extracted the following data from included studies using a pre-piloted standardised form [59, 60]: study design, participant characteristics, modifiable risk factor/s, implementation strategy/ies, comparison group, care element/s targeted by implementation strategy/ies, primary and secondary outcomes, theoretical basis, process implementation measures and information to assess risk of bias. Implementation strategies were classified according to the EPOC Taxonomy (see Table 1) [35]. Discrepancies in data extraction were resolved by consensus, or with a third reviewer.

#### Assessment of risk of bias

Two review authors (SM and one of MK and JH) independently assessed the risk of bias of included studies. For randomised studies, the Cochrane Risk of Bias Tool (RoB 1) [54] was used to assess: random sequence generation, allocation concealment, blinding of participants and personnel, blinding of outcome assessments, incomplete outcome data, selective outcome reporting, and any other potential sources of bias. For cluster RCTs, additional criteria included recruitment to cluster, baseline imbalance, loss of clusters, incorrect analysis, and compatibility with individual RCTs [54]. For non-randomised studies, the Newcastle-Ottawa Scale (NOS) [61] was used: selection, comparability, and outcome. Discrepancies regarding the assessment of bias were resolved by consensus, or with a third reviewer.

#### Assessment of the certainty of evidence

Two review authors (ED, SM) independently assessed the certainty of the evidence for each primary outcome synthesised in a meta-analysis using the Grading of Recommendations, Assessment, Development, and Evaluation (GRADE) approach [62]. Domains assessed included risk of bias, inconsistency, indirectness, imprecision, and publication bias. Discrepancies in GRADE assessments were resolved by consensus, or with a third reviewer.

#### Data analysis and synthesis

As per the Cochrane Handbook [54], meta-analyses were conducted when outcome measures from at least two studies could be pooled using random-effects models. Primary outcomes were synthesised separately for RCTs and non-randomised studies, by modifiable risk factor (tobacco smoking, weight management, alcohol consumption), recommended care element (tobacco smoking: ask, advise, assess, assist, arrange; weight management/alcohol consumption: assess, advise, refer) and comparator type (usual practice/control, alternative implementation strategies). Dichotomous data was pooled and treatment effects were expressed as odds ratios (ORs) using reported effect estimates when available or between group data [63]. Results presented in other formats (e.g. means and standard deviations) were transformed to ORs and 95% Confidence Intervals (CIs) where possible to enable pooling [64]. Studies that reported multiple results for the same outcome (e.g. two components of recommended advice) from the same sample of participants, were combined to create one summary effect for each outcome using recommended formulas [63, 65]. In these analyses, to account for the non-independence of multiple results relating to the same outcome, we assumed a correlation coefficient of 0.7 when calculating the standard error of the summary effect. Sensitivity analyses were conducted with correlation coefficients of 0.8 and 0.9 to test this assumption against the robustness of the findings.

Secondary outcomes were similarly synthesised separately for RCTs and non-randomised studies, and by modifiable risk factors. Outcome data were pooled as either ORs for dichotomous or mean differences for continuous data and 95% CIs reported.

The *I*^2^ statistic for each pooled result was calculated to assess statistical heterogeneity. The *I*^2^ statistic estimates the proportion of variance in a meta-analysis that is attributable to heterogeneity rather than chance. The value of the *I*^2^ statistic ranges between 0% to 100% with higher percentages indicating higher heterogeneity [54]. Unit of analysis errors in cluster trials was examined, and where identified study data was used to calculate design effects and effective sample sizes based on Cochrane guidance [54]. In instances where there was heterogeneity in the comparison group, results from an individual study could not be pooled, or only one study contributed results, findings were summarised using the direction of effect [66].

## Results

### Study selection

**Fig. 1:**
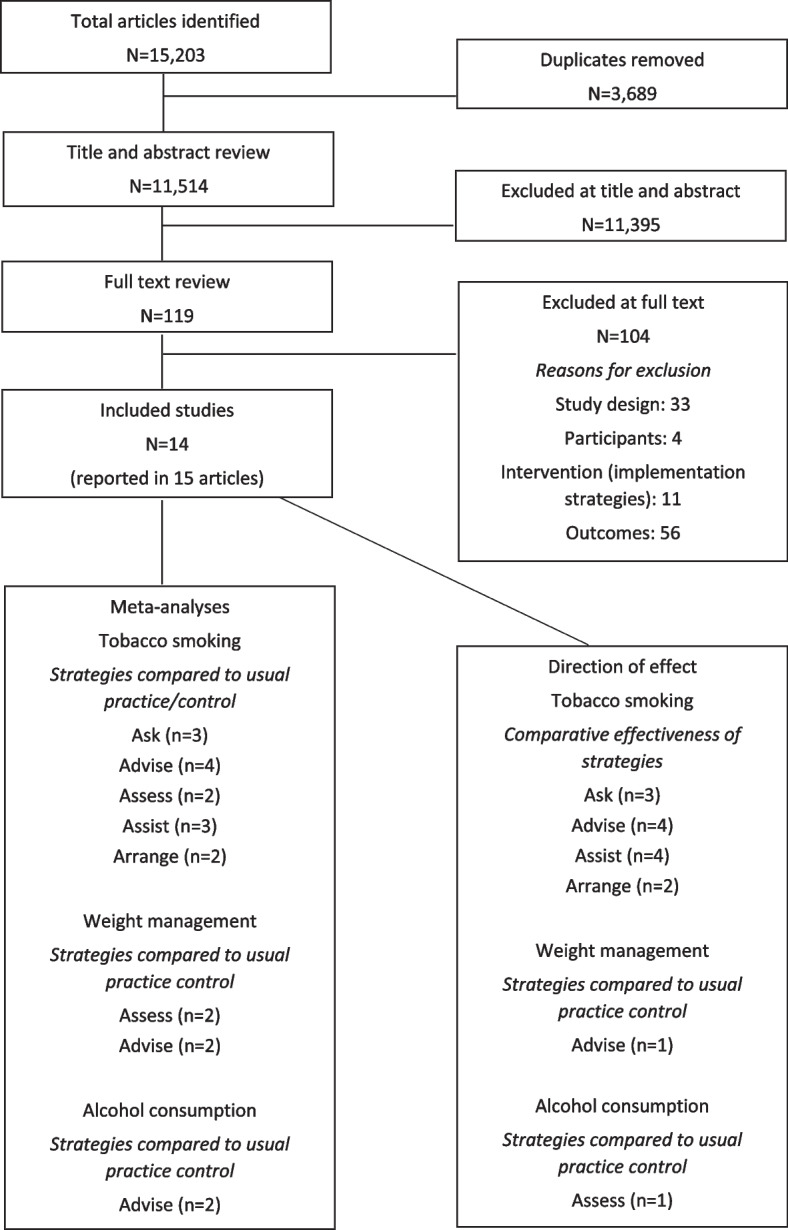
PRISMA flow diagram

The search identified 15,203 records (see Fig. 1). After duplicates were removed, titles and abstracts of 11,514 records were screened, of which 119 were sought for full-text review. No articles were excluded at full-text due to an English translation not being able to be sourced. Fourteen studies reported in 15 articles met eligibility criteria and were included (see Supplementary File 3 for characteristics of included studies).

### Included studies

#### Types of studies

Included studies were published between 1992 and 2020. Ten were RCTs [51–53, 67–74], of which four were cluster-RCTs [51, 52, 71, 72], and four were non-randomised controlled trials [49, 50, 75, 76]. Studies were conducted in 10 countries: four in the United States [49, 72–74], three in Australia [52, 53, 69, 70] and one each in the United Kingdom [68], Italy [75], The Netherlands [67], South Africa [76], Ethiopia [51], Brazil [50], and Argentina and Uruguay [71].

#### Participants

One study focussed on care provision during the preconception period [76] and 13 during the antenatal period [49–53, 67–75]. Seven studies were conducted in primary/community-based services [50–52, 67, 73, 74, 76], four in hospital-based services [49, 53, 69, 70, 75] and three both [68, 71, 72]. The number of services in studies ranged from one [53, 73] to 42 [67]. Health professionals targeted by the implementation strategies included: multidisciplinary teams (*n*=8; doctors, nurses, midwives, Aboriginal Health Workers) [49–52, 69–72, 75], midwives only (*n*=2) [67, 68], doctors only (*n*=1) [73] and public sector workers (*n*=1) [74]. Two studies did not specify the discipline of the health professional providing care [53, 74].

#### Interventions (implementation strategies)

A single implementation strategy was tested in two studies [74, 75] and multiple strategies in the remaining 12 studies [49–53, 67–73, 76] (median: three; range: two to five). Nine types of implementation strategies were assessed by the included studies, with educational meetings (*n*=12) [49–52, 67–73, 75, 76], educational materials (*n*=10) [49–53, 67–70, 72, 76], reminders (*n*=5) [53, 68, 71, 73, 74], educational outreach visits or academic detailing (*n*=4) [51, 69–72] and tailored interventions (*n*=4) [52, 69–71] the most commonly assessed (see Table 1). Five studies reported the use of a model, theory, or framework in strategy development (either Theoretical Domains Framework and Behaviour Change Wheel [52] or Roger’s Diffusion of Innovation Theory [69–72]). Seven studies reported on at least one measure of the implementation process [49, 51, 52, 67–71], with fidelity (*n*=5) [51, 52, 67, 70, 71] and acceptability (*n*=3) [49, 52, 67] the most commonly reported.

**Table 1:** Implementation strategies (EPOC Taxonomy) used in the intervention group of included studies

Implementation strategy	Definition	Number of studies tested in
Educational meetings	Courses, workshops, conferences or other educational meetings.	12 [49–52, 67–73, 75, 76]
Educational materials	Distribution to individuals, or groups, of educational materials to support clinical care, i.e. any intervention in which knowledge is distributed.	10 [49–53, 67–70, 72, 76]
Reminders	Manual or computerised interventions that prompt health workers to perform an action during a consultation with a patient, for example computer decision support systems.	5 [53, 68, 71, 73, 74]
Educational outreach visits or academic detailing	Personal visits by a trained person to health workers in their own settings, to provide information with the aim of changing practice.	4 [51, 69–72]
Tailored interventions	Interventions to change practice that are selected based on an assessment of barriers to change, for example through interviews or surveys.	4 [52, 69–71]
Clinical practice guidelines	Systematically developed statements to assist healthcare providers and patients to decide on appropriate health care for specific clinical circumstances.	3 [52, 67, 72]
Audit and feedback	A summary of health workers’ performance over a specified period of time, given to them in a written, electronic or verbal format. The summary may include recommendations for clinical action.	1 [69, 70]
Local opinion leaders	The identification and use of identifiable local opinion leaders to promote good clinical practice.	1 [71]
Local consensus process	Formal or informal local consensus processes, for example agreeing a clinical protocol to manage a patient group, adapting a guideline for a local health system or promoting the implementation of guidelines.	1 [50]
Clinical incident reporting	System for reporting critical incidents.	0
Communities of practice	Groups of people with a common interest who deepen their knowledge and expertise in this area by interacting on an ongoing basis.	0
Continuous quality improvement	An iterative process to review and improve care that includes involvement of healthcare teams, analysis of a process or system, a structured process improvement method or problem-solving approach, and use of data analysis to assess changes.	0
Educational games	The use of games as an educational strategy to improve standards of care.	0
Inter-professional education	Continuing education for health professionals that involves more than one profession in joint, interactive learning.	0
Managerial supervision	Routine supervision visits by health staff.	0
Monitoring the performance of the delivery of healthcare	Monitoring of health services by individuals or healthcare organisations, for example by comparing with an external standard.	0
Patient mediated interventions	Any intervention aimed at changing the performance of healthcare professionals through interactions with patients, or information provided by or to patients.	0
Public release of performance data	Informing the public about healthcare providers by the release of performance data in written or electronic form.	0
Routine patient-reported outcome measures	Routine administration and reporting of patient reported outcome measures to providers and/or patients.	0

#### Comparisons

Ten studies compared implementation strategies to usual practice or a control condition [49–53, 67, 68, 74–76] and four compared different combinations of strategies [69–73].

#### Outcomes

##### Primary outcomes — provision of recommended care

Eight studies sought to improve care addressing smoking [52, 67–74], four weight management [49–51, 53] and two alcohol consumption [75, 76]. Outcomes were assessed using women’s self-report (*n*=9) [50, 68, 70–76], health professional self-report (*n*=2) [52, 69], both women’s and health professional’s self-report (*n*=2) [49, 67], direct observations (*n*=1) [51] and medical record audit (*n*=1) [53].

##### Secondary outcomes

Four studies reported the effects of implementation strategies on women’s smoking [68, 70, 71, 74], two on weight gain [49, 53] and none on nutrition, physical activity or alcohol consumption. No studies reported estimates of absolute costs or cost-effectiveness of the implementation strategies, or any unintentional adverse consequences.

### Risk of bias in included studies

Of the 10 RCTs, random sequence generation resulted in low risk of bias in six studies [51, 53, 67, 68, 71, 74] and risk was unclear in four [52, 69, 70, 72, 73]. The processes of allocation concealment was assessed as unclear in six studies [67–73], low risk in three [51, 53, 74] and high risk in one [52]. All studies were assessed as high risk for performance bias, with none reporting blinding of both participants and personnel. Blinding of outcome assessment was unclear for the majority of studies (*n*=7) [51, 53, 68, 71–74]. Incomplete reporting of outcome data was rated as high risk in three studies [52, 67, 69, 70], low in six [51, 53, 68, 71, 73, 74] and unclear in one [72]. The presence of reporting bias was unclear for most studies (*n*=8) [51, 53, 67–70, 72–74]. Two studies were assessed as being at high risk of bias due to contamination [73, 74]. Of the four cluster RCTs [51, 52, 71, 72], there was a high risk of bias in two studies for loss of clusters [52, 72] and in one study for recruitment to cluster [52] and incorrect analysis [72] (see Table 2).

**Table 2:** Summary of risk of bias for randomised studies

Study	Criteria for judging risk of bias for RCTs						Additional criteria for cluster RCTs					Other
	Random sequence generation	Allocation concealment	Blinding of participants and personnel	Blinding of outcome assessment	Incomplete outcome data	Selective reporting	Recruitment to cluster	Baseline imbalance	Loss of clusters	Incorrect analysis	Compatibility with individual RCTs	
Althabe et al. (2017) [71]	Low	Unclear	High	Unclear	Low	Low	Low	Low	Low	Low	Low	-
Bakker et al. (2003) [67]	Low	Unclear	High	High	High	Unclear	NA	NA	NA	NA	NA	-
Bar-Zeev et al. (2019) [52]	Unclear	High	High	High	High	Low	High	Unclear	High	Low	Unclear	-
Brownfoot et al. (2016) [53]	Low	Low	High	Unclear	Low	Unclear	NA	NA	NA	NA	NA	-
Campbell et al. (2006) [70] and Cooke et al. (2001) [69]	Unclear	Unclear	High	High	High	Unclear	NA	NA	NA	NA	NA	-
Hajek et al. (2001) [68]	Low	Unclear	High	Unclear	Low	Unclear	NA	NA	NA	NA	NA	-
Manfredi et al. (2011) [72]	Unclear	Unclear	High	Unclear	Unclear	Unclear	Low	Unclear	High	High	Unclear	-
Omer et al. (2020) [51]	Low	Low	High	Unclear	Low	Unclear	Low	Low	Low	Low	Unclear	-
Secker-walker et al. (1992) [73]	Unclear	Unclear	High	Unclear	Low	Unclear	NA	NA	NA	NA	NA	High^a^
Tsoh et al. (2010) [74]	Low	Low	High	Unclear	Low	Unclear	NA	N/A	NA	NA	NA	High^a^

^a^High risk of contamination

In relation to selection bias in non-randomised studies, all of the studies included a representative sample in the intervention group [49, 50, 75, 76] and in three studies the control group/s was drawn from the same service type as the intervention group/s [49, 75, 76] (see Table 3). However, three studies did not provide a description of the response rate or characteristics of responders and non-responders [49, 75, 76]. Only two studies indicated the outcome of interest at the start of the study [49, 76]. Comparability of intervention and control groups was a source of bias based on study design [50, 75] and analysis that did not control for confounding factors [49, 50, 75, 76]. Considerable risk of bias was introduced in relation to outcomes. None of the studies used independent blind assessment or self-report of participants who were blind to allocation. Only two studies reported an appropriate statistical test to analyse the data and presented the outcome measurement (e.g. confidence intervals and/or *p*-value) [75, 76].

**Table 3:** Summary of risk of bias for non-randomised studies

Study	Selection				Comparability	Outcome			Total score
	Representativeness of the sample in the intervention group/s: Max: ٭	Selection of the control group/s: Max: ٭	Non-respondents: Max: ٭	Demonstration of outcome of interest at start of study: Max: ٭	Comparability of intervention and control groups on the basis of the design or analysis Max:٭٭	Assessment of outcome: Max: ٭	Follow-up long enough for the outcome of interest to occur: Max: ٭	Statistical test for outcome of interest: Max: ٭	Total score (out of 9)
Aguilera et al. (2017) [49]	٭	٭	-	٭	٭	-	-	-	٭٭٭٭ (4)
Bazzo et al. (2015) [75]	٭	٭	-	-	-	-	٭	٭	(4) ٭٭٭٭
Malta et al. (2016) [50]	٭	-	٭	-	-	-	٭	-	(3) ٭٭٭
Mwansa-Kambafwile et al. (2011) [76]	٭	٭	-	٭	٭	-	٭	٭	(6) ٭٭٭٭٭٭

Based on a star system (*) with a range of 0 to 9 stars possible. Three domains are tested: (1) selection of study groups (up to one star allowed for each item), (2) comparability of the groups (up to two stars allowed), and (3) outcomes (up to one star allowed for each item) [61]

### Effect of implementation strategies in improving the provision of recommended preconception and antenatal care addressing modifiable risk factors

Table 4 provides an overview of the synthesis and included studies, and Table 5 the pooled effect estimates and GRADE assessments.

**Table 4:** Overview of Synthesis and Included Studies (OSIS)

Study ID	Study design	Preconception/antenatal care	Participants	Implementation strategies	Comparator	Outcomes	Method of synthesis
**Tobacco smoking**							
Bakker et al. (2003) [67]	RCT	Antenatal care	Private midwifery practices Midwives	Clinical practice guidelines Educational meetings Educational materials	Usual practice/control	Ask Advise Assess Assist Arrange	Meta-analysis Meta-analysis Meta-analysis Meta-analysis Meta-analysis
Bar-Zeev et al. (2019) [52]	Cluster RCT	Antenatal care	Aboriginal Medical Services General Practitioners, midwives, Aboriginal Health Workers and other allied health providers	Clinical practice guidelines Educational meetings Educational materials Tailored intervention	Usual practice/control	Ask Advise Assess Assist Arrange	Meta-analysis Meta-analysis Meta-analysis Meta-analysis Meta-analysis
Hajek et al. (2001) [68]	RCT	Antenatal care	Midwifery services in hospital and community trusts Midwives	Educational meetings Educational materials Reminders	Usual practice/control	Ask Advise Assist	Meta-analysis Meta-analysis Meta-analysis
Tsoh et al. (2010) [74]	RCT	Antenatal care	Community prenatal clinics Prenatal healthcare providers	Reminders	Usual practice/control	Advise	Meta-analysis
Althabe et al. (2017) [71]	Cluster RCT	Antenatal care	Antenatal care clinics Midwives and obstetrician/gynaecologists	Educational meetings Educational outreach visits, or academic detailing Local opinion leaders Reminders Tailored intervention	Educational meetings	Ask Advise Assist Arrange	Direction of effect Direction of effect Direction of effect Direction of effect
Campbell et al. (2006) [70]; Cooke et al. (2001) [69]	RCT	Antenatal care	Public hospital antenatal clinics Doctors and midwives	Audit and feedback Educational materials Educational meetings Educational outreach visits, or academic detailing Tailored intervention	Educational materials	Ask Advise Assist Arrange	Direction of effect Direction of effect Direction of effect Direction of effect
Secker-walker et al. (1992) [73]	RCT	Antenatal care	Maternal infant care clinic Obstetric and family practice residents	Educational meetings Reminders	Educational meetings	Ask Advise Assist	Direction of effect Direction of effect Direction of effect
Manfredi et al. (2011) [72]	Cluster RCT	Antenatal care	Maternal and child health public health clinics Doctors and nurses	Clinical practice guideline Educational materials Educational meetings Educational outreach visits, or academic detailing	Clinical practice guideline Educational materials Educational meetings	Advise Assist	Direction of effect Direction of effect
**Weight management**							
Brownfoot et al. (2016) [53]	RCT	Antenatal care	Antenatal clinics in a tertiary obstetric hospital Antenatal care providers	Educational materials Reminders	Usual practice/control	Assess	Meta-analysis
Omer et al. (2020) [51]	Cluster RCT	Antenatal care	Antenatal care units in community health centres Health officers, nurses and midwives	Educational materials Educational meetings Educational outreach visits, or academic detailing	Usual practice/control	Assess Advise	Meta-analysis Direction of effect
Aguilera et al. (2017) [49]	Non-randomised	Antenatal care	Obstetrics practices Physicians and nurses	Educational meetings Educational materials	Usual practice/control	Advise	Meta-analysis
Malta et al. (2016) [50]	Non-randomised	Antenatal care	Primary care services and family health units Doctors and nurses	Educational materials Educational meetings Local consensus process Tailored intervention	Usual practice/control	Advise	Meta-analysis
**Alcohol consumption**							
Bazzo et al. (2015) [75]	Non-randomised	Antenatal care	Hospital obstetrics and gynaecology units Midwives	Educational meetings	Usual practice/control	Advise	Meta-analysis
Mwansa-Kambafwile et al. (2011) [76]	Non-randomised	Preconception care	Public healthcare services Public sector healthcare workers	Educational materials Educational meetings	Usual practice/control	Assess Advise	Direction of effect Meta-analysis

**Table 5:** Effect of implementation strategies in improving the provision of preconception and antenatal care addressing modifiable risk factors

Outcome	Study design	Implementation strategies	Comparator	Meta-analysis OR (95% CI; *p*)	I^**2**^	Certainty of the evidence	Results of studies not in meta-analysis
**Tobacco smoking**							
Ask	RCT	Clinical practice guidelines; educational materials; educational meetings; reminders; tailored intervention	Usual practice/control	2.52 (1.13, 5.59; *p*=0.024)	47%	Moderate^c^	-
Advise	RCT	Clinical practice guidelines; educational materials; educational meetings; reminders; tailored intervention	Usual practice/control	4.32 (3.06, 6.11; *p*<0.001)	0%	Moderate^c^	-
Assess	RCT	Clinical practice guidelines; educational materials; educational meetings; tailored intervention	Usual practice/control	2.55 (0.24, 27.06; *p*=0.439)	90%	Low^c,d,e^	-
Assist	RCT	Clinical practice guidelines; educational materials; educational meetings; reminders; tailored intervention	Usual practice/control	6.34 (1.51, 26.63; *p*=0.012)	90%	Low^c,d^	-
Arrange	RCT	Clinical practice guidelines; educational materials; educational meetings; tailored intervention	Usual practice/control	3.55 (0.50, 25.34; *p*=0.207)	83%	Low^c,d,e^	-
Ask	RCT	Audit and feedback; educational materials; educational meetings; educational outreach visits, or academic detailing; tailored intervention	Educational materials	-	-	-	1.2 (1.0, 1.5)^a^
		Educational meetings; reminders	Educational meetings	-	-	-	6.3 (1.8, 22.1)^a^
		Educational meetings; educational outreach visits, or academic detailing; local opinion leaders; reminders; tailored intervention	Educational meetings	-	-	-	29.2 (17.5, 38.0)^b^
Advise	RCT	Audit and feedback; educational materials; educational meetings; educational outreach visits, or academic detailing; tailored intervention	Educational materials	-	-	-	1.1 (0.9, 1.3)^a^
		Educational meetings; reminders	Educational meetings	-	-	-	4.5 (1.9, 10.8)^a^
		Educational meetings; educational outreach visits, or academic detailing; local opinion leaders; reminders; tailored intervention	Educational meetings	-	-	-	26.2 (13.9, 40.2)^b^
		Clinical practice guideline; educational materials; educational meetings; educational outreach visits, or academic detailing	Clinical practice guideline; educational materials; educational meetings	-	-	-	1.95 (1.32, 2.88)^a^
Assist	RCT	Audit and feedback; educational materials; educational meetings; educational outreach visits, or academic detailing; tailored intervention	Educational materials	-	-	-	1.3 (0.9, 1.9)^a^
		Educational meetings; reminders	Educational meetings	-	-	-	29.9 (14.5, 61.9)^a^
		Educational meetings; educational outreach visits, or academic detailing; local opinion leaders; reminders; tailored intervention	Educational meetings	-	-	-	21.5 (10.6, 31.8)^b^
		Clinical practice guideline; educational materials; educational meetings; educational outreach visits, or academic detailing	Clinical practice guideline; educational materials; educational meetings	-	-	-	1.96 (1.13, 3.39)^a^
Arrange	RCT	Audit and feedback; educational materials; educational meetings; educational outreach visits, or academic detailing; tailored intervention	Educational materials	-	-	-	1.07 (0.57, 1.99)^a^
		Educational meetings; educational outreach visits, or academic detailing; local opinion leaders; reminders; tailored intervention	Educational meetings	-	-	-	2.7 (0.0, 17.2)^b^
**Weight management**							
Assess	RCT	Educational materials; educational meetings; educational outreach visits, or academic detailing; reminders	Usual practice/control	57.56 (41.78, 79.29; *p*<.001)	0%	Moderate^c^	-
Advise	RCT	Educational materials; educational meetings; educational outreach visits, or academic detailing	Usual practice/control	-	-	-	6.44 (3.14, 13.17)^a^
Advise	Non-randomised	Educational materials; educational meetings; local consensus process; tailored intervention	Usual practice/control	3.37 (2.34, 4.84; *p*<0.001)	0%	Very-low^c,d^	-
**Alcohol consumption**							
Assess	Non-randomised	Educational materials; educational meetings	Usual practice/control	-	-	-	1.15 (0.17, 1.03)^a^
Advise	Non-randomised	Educational materials; educational meetings	Usual practice/control	10.36 (2.37, 41.20; *p*=0.002)	83%	Very-low^c,f^	-

^a^Odds ratio (95% CI); ^b^absolute difference in medians (95% CI)

Reasons for downgrading certainty of the evidence ratings: ^c^risk of bias; ^d^inconsistency; ^e^imprecision; ^f^indirectness

#### Tobacco smoking

##### Implementation strategies compared to usual practice/control

*Ask:* Three RCTs [52, 67, 68] examined the effect of implementation strategies in supporting health professionals to ask about tobacco smoking during pregnancy compared to usual practice/control. Meta-analysis of these studies found a significant positive effect (OR: 2.52; 95% CI: 1.13, 5.59; *p*=0.024; *I*^2^: 47%; moderate-certainty evidence). The studies tested a combination of either three [67, 68] or four strategies [52], and all included educational meetings and educational materials [52, 67, 68]. Clinical practice guidelines were tested by two studies [52, 67], and reminders [68] and a tailored intervention [52] in one study each.

*Advise:* Four RCTs [52, 67, 68, 74] examined the effect of implementation strategies in supporting health professionals to provide pregnant women with advice to quit smoking compared to usual practice/control. Meta-analysis of these studies found a significant positive effect (OR: 4.32; 95% CI: 3.06, 6.11; *p*<0.001; *I*^2^: 0%; moderate-certainty evidence). Three of the studies were multi-strategy [52, 67, 68], of which all used educational meetings and educational materials, and in addition some also included clinical practice guidelines [52, 67], reminders [68] and/or tailored intervention [52]. One study used a single strategy of reminders [74].

*Assess:* Two RCTs [52, 67] examined the effect of implementation strategies in supporting health professionals to assess women’s willingness to quit smoking compared to usual practice/control. Meta-analysis of these studies found higher odds of assessment in the intervention group, although the result was not significant (OR: 2.55; 95% CI: 0.24, 27.06; *p*=0.439; *I*^2^: 90%; low-certainty evidence). Both studies used multiple implementation strategies, including clinical practice guidelines, educational meetings and educational materials [52, 67], with one study also using tailored intervention [52].

*Assist:* Three RCTs [52, 67, 68] examined the effect of implementation strategies in supporting health professionals to assist pregnant women with quitting smoking compared to usual practice/control. Meta-analysis of these studies found a significant positive effect (OR: 6.34; 95% CI: 1.51, 26.63; *p*=0.012; *I*^2^: 90%; low-certainty evidence). The studies tested a combination of three [67, 68] or four strategies [52], and all included educational meetings and educational materials [52, 67, 68]. Clinical practice guidelines were used by two studies [52, 67] and reminders [68], and tailored intervention [52] in one study each.

*Arrange:* Two RCTs [52, 67] examined the effect of implemention strategies in supporting health professionals to arrange support for smoking cessation compared to usual practice/control. Meta-analysis of these studies found higher odds of arranging support in the intervention group; however, this was not significant (OR: 3.55; 95% CI: 0.50, 25.34; *p*=0.207; *I*^2^: 83%; low-certainty evidence). Both studies used multiple implementation strategies that included clinical practice guidelines, educational meetings and educational materials [52, 67], with one study also using tailored intervention [52].

##### Comparative effectiveness of implementation strategies

*Ask, Advise, Assist and Arrange:* Four RCTs [69–73] examined the comparative effectiveness of implementation strategies in supporting health professionals to provide recommended tobacco smoking care, which were unable to be synthesised in meta-analysis. The first of these studies compared five implementation strategies (audit and feedback, educational materials, educational meetings, educational outreach visits and tailored intervention) to educational materials and found a positive direction of effect for asking (OR: 1.2; 95% CI: 1.0, 1.5), advising (OR: 1.1; 95% CI: 0.9, 1.3), assisting (OR: 1.3; 95% CI: 0.9, 1.9) and arranging follow-up (OR: 1.07; 95% CI: 0.57, 1.99) [70]. The second study, which examined the effect of educational meetings and reminders compared to educational meetings found a positive direction of effect for asking (OR: 6.3; 95% CI: 1.8, 22.1), advising (OR: 4.5; 95% CI: 1.9, 10.8) and assisting (OR: 29.9; 95% CI: 14.5, 61.9) [69, 73]. The third study tested the effect of five implementation strategies (educational meetings, educational outreach visits, local opinion leaders, reminders and tailored intervention) compared to a single strategy of educational meetings and found a positive direction of effect for asking (difference in medians (DIM): 29.2; 95% CI: 17.5, 38.0); advising (DIM: 26.2; 95% CI: 13.9, 40.2); assisting (DIM: 21.5; 95% CI: 10.6, 31.8) and arranging follow-up (DIM: 2.7; 95% CI: 0, 17.2) [71]. The last study examined the effect of clinical practice guidelines, educational materials, educational meetings and educational outreach visits compared to all the same strategies other than educational outreach visits [72]. A positive direction of effect was reported for advice (OR: 1.95; 95% CI: 1.32, 2.88) and assistance with quitting smoking (OR: 1.96; 95% CI: 1.13, 3.39).

#### Weight management

##### Implementation strategies compared to usual practice/control

*Assess:* Two RCTs [51, 53] examined the effect of implementation strategies in supporting health professionals to assess pregnant women’s weight gain within recommendations compared to usual practice/control. Meta-analysis of these studies found a significant positive effect (OR: 57.56; 95% CI: 41.78, 79.29; *p*<0.001; *I*^2^: 0%; moderate-certainty evidence). Both studies tested multiple implementation strategies, with one using educational materials and reminders [53] and the other educational materials, educational meetings and educational outreach visits [51].

*Advise:* Three studies (one RCT [51] and two non-randomised [49, 50]) examined the effect of implementation strategies in supporting health professionals to advise pregnant women about weight gain recommendations compared to usual practice/control [49–51]. The RCT [51] used educational materials, educational meetings and educational outreach visits and reported a positive direction of effect (OR: 6.44; 95% CI: 3.14, 13.17). Meta-analysis of the two non-randomised studies found a significant positive effect (OR: 3.37; 95% CI: 2.34, 4.84; *p*<0.001; *I*^2^: 0%; very low-certainty evidence). Both studies tested a combination of educational materials and educational meetings [49, 50], with one study using additional strategies of local consensus process and tailored intervention [50].

#### Alcohol consumption

##### Implementation strategies compared to usual practice/control

*Assess:* One non-randomised study [76] examined the effect of multiple implementation strategies in supporting health professionals to assess alcohol consumption in preconception care compared to usual practice/control. The study used educational materials and educational meetings and reported a positive direction of effect (OR: 1.15, 95% CI: 0.17, 1.03) [76].

*Advise:* Two non-randomised studies [75, 76] examined the effect of implementation strategies in supporting health professionals to provide preconception and antenatal advice not to consume alcohol compared to usual practice/control. Meta-analysis of these two studies found a significant positive effect (OR: 10.36; 95% CI: 2.37, 41.20; *I*^2^: 83%; very low-certainty evidence). The study in the preconception period used educational meetings and educational materials [76], and the one in antenatal used educational meetings [75].

No studies reported on the effect of implementation strategies in supporting health professionals provide tobacco smoking and weight management care or referrals for alcohol consumption during the preconception period. There were no studies reporting on increasing referrals for weight management and alcohol consumption in antenatal care.

### Secondary outcomes

#### Effect of implementation strategies on pregnant women’s modifiable risk factors

Four RCTs [68, 70, 71, 74] examined the effect of implementation strategies on quit smoking outcomes during pregnancy. Meta-analysis of these studies found that implementation strategies to improve care practices significantly increased the odds of cessation by 43% (OR: 1.43; 95% CI: 1.09, 1.88; *p*=0.009; *I*^2^: 16%). The four studies tested either a single (reminders) [74] or a combination of multiple implementation strategies (audit and feedback, educational materials, educational meetings, educational outreach visits, local opinion leaders, reminders and tailored intervention) [68, 70, 71].

Two studies [49, 53] reported on pregnant women’s weight gain within recommendations, with no effect found in either the RCT that used educational materials and reminders (OR: 1.04; 95% CI: 0.67, 1.64) [53] or the non-randomised trial that used educational materials and educational meetings (OR: 0.99; 95% CI: 0.63, 1.55) [49]. The RCT also reported no effect in weight gained per week between intervention and control groups (mean difference: 0.01; 95% CI: −0.03, 0.05) [53].

#### Implementation costs/cost-effectiveness and unintentional adverse consequences

No studies reported estimates of the absolute costs or cost-effectiveness or unintentional adverse consequences of the implementation strategies.

## Discussion

This systematic review examined the effectiveness of implementation strategies in improving health professional’s provision of guideline-recommended preconception and antenatal care addressing tobacco smoking, weight management and alcohol consumption. Meta-analyses combined with GRADE assessments to assess the certainty of the evidence indicated that implementation strategies probably increase asking and advising about smoking and assessing weight gain in pregnancy compared to usual practice/control, and may increase assessing, assisting and arranging support for smoking. Whilst the pooled effect estimates for weight gain and alcohol consumption advice were also in a positive direction, the certainty of the evidence was assessed as very-low, implying that the true effects are not known. There was a positive direction of effect for multiple implementation strategies versus single strategy (either educational meetings or materials) in improving smoking care. Meta-analyses of modifiable risk factor outcomes found increased odds of quitting smoking, though no improvements in pregnant women’s gestational weight gain.

The finding that implementation strategies probably increase elements of smoking and weight management antenatal care is consistent with broader Cochrane systematic review evidence regarding the effect of implementation strategies in healthcare settings [36–42]. All but one of the studies contributing data to these outcomes tested multiple implementation strategies, with a median of three strategies used. Strategies for improving asking and advising about smoking included educational materials, educational meetings, reminders, clinical practice guidelines and tailored intervention. Similarly, educational materials, educational meetings and reminders were used to increase assessment of weight gain, with the addition of educational outreach visits. These strategies are consistent with the previous antenatal care smoking review [47], which found that three or more implementation strategies, theoretical/tailored strategy development and inclusion of a systems-based strategy were among the components of implementation that may have led to a positive impact. Such review findings support policymaker and health service adoption of multiple implementation strategies, such as educational materials, educational meetings and reminders, to increase the provision of recommended smoking care and weight gain assessment to pregnant women by health professionals.

There was a positive direction of effect for multiple implementation strategies versus a single strategy in supporting health professionals deliver recommended smoking care to pregnant women. Further, two of the three studies specifically selected implementation strategies that targeted barriers reported by health professionals [69–71], which is a recommended step in the design of implementation trials [77]. Frequently clinical guidelines released by government or professional bodies are simply accompanied by a single strategy, such as the distribution of educational materials (e.g. health professional handouts) or education (e.g. online training module) [78]. However, previous research has shown that selecting implementation strategies that target the specific barriers cited by health professionals can increase various evidence-based care practices by over 50% [79]. Such findings suggest clinical guideline concordant care for pregnant women could be maximised if multiple implementation strategies are used, including those that target the specific barriers cited by health professionals.

This review highlighted gaps in the evidence-base for how to improve preconception and antenatal care to address modifiable risk factors. Whilst the pooled effect estimates for weight gain and alcohol consumption advice were positive, the very-low certainty of evidence ratings implies that new studies in this area could substantially change the estimate. Further, no studies were identified that examined the effect of implementation strategies in improving any element of preconception care addressing smoking and weight management, or referrals for alcohol consumption and weight management during the antenatal period. Given the critical importance of women entering pregnancy in optimal health and being supported to modify their risk factors during pregnancy, priority research is required to inform implementation strategies for these specific care elements. Furthermore, there was a lack of information across all included studies regarding the cost and unintended adverse consequences of implementation strategies. Such information, which is essential for guiding policy and practice decision-making and investment [80], should be assessed and reported in future studies.

The findings of this review need to be considered in light of a number of strengths and limitations. The review adopted best practice systematic review methods and employed a broad inclusion criteria, which enabled a comprehensive synthesis of the evidence-base. However, there is potential that eligible articles were missed as the search was conducted in English only and articles without an English translation available were made ineligible. This may have contributed to the small number of studies found in low and middle-income countries, and without such representation, the external validity of the review findings is largely limited to high-income countries. The generalisability of the review findings to preconception care is also limited as only one study was identified in this setting. There is currently no agreed definition of what constitutes the preconception population in the literature base [81], and this, as well as the more restricted definition used in this review, may have contributed to the lack of studies that were identified. The review also restricted eligible study designs to those that had a parallel comparison group. This omitted non-control studies that may have provided further information useful for understanding implementation strategies used in improving guideline-recommended care addressing modifiable risk factors. Reviewer interpretation was used to classify strategies to the EPOC taxonomy, which may have introduced variability during synthesis due to the inconsistencies in the terminology used across studies. It is possible that strategies not incorporated within the EPOC taxonomy were also missed through this process.

The interpretation and utility of the review findings were limited by the characteristics of the included studies. No studies for any of the risk factors tested the same combination of strategies, which prohibited the examination of specific individual strategies and strategy combinations. Further, there was heterogeneity in the types of services (e.g. hospital and community based) and health professional groups (e.g. midwives and medical staff) targeted by the implementation strategies in the included studies, and two studies did not not specify the discipline of the health professionals who participated. Such heterogeneity and missing data, as well as the small number of included studies, prohibited synthesis by the distinct service and health professional groupings.

Meta-analysis was not possible for all outcomes, and where possible, only a small number of studies (2 to 4) were able to be synthesised, which is likely to have impacted on the ability of the random effects meta-analyses to reliably estimate the between-study variation. As per recommendations for synthesising results from implementation trials in healthcare settings [54, 82], non-randomised study designs were included. However, as only non-randomised studies were found that examined the effect of implementation strategies in improving alcohol consumption care, this limits certainty in the evidence-base for these specific outcomes.

## Conclusions

Review findings suggest the adoption of multiple implementation strategies, including educational materials, educational meetings and reminders, by policy makers and health services to increase health professional provision of asking and advising about tobacco smoking and assessing weight gain in pregnancy. Rigorous research is needed as a priority to build certainty in the evidence for improving alcohol consumption and weight gain advice during the antenatal period and to examine the effect of implementation strategies in preconception care where limited studies were identified.

## Supplementary Information

**Additional file 1.** PRISMA Checklist.

**Additional file 2.** Search Strategy.

**Additional file 3.** Characteristics of Included Studies.

## Data Availability

All articles included in this systematic review are publicly available. The datasets used and/or analysed during the current study are available from the corresponding author on reasonable request.

## Abbreviations

95% CI: Ninety-five per cent confidence interval
DIM: Difference In Medians
EPOC: Effective Practice and Organisation of Care
GRADE: Grading of Recommendations, Assessment, Development, and Evaluation
OR: Odds ratio
OSIS: Overview of Synthesis and Included Studies
RCT: Randomised controlled trial
RoB: Risk of bias
