# Supplementary material for: The effectiveness of implementation strategies in improving preconception and antenatal preventive care: a systematic review
Source: Implement Sci Commun. 2022 Nov 22;3:121. doi: 10.1186/s43058-022-00368-1 (PMC9682815; doi:10.1186/s43058-022-00368-1)
Supplement: Supplementary file 2 — Additional file 2. Search Strategy. [file 43058_2022_368_MOESM2_ESM.docx]

**Supplementary File 2.** Search Term Strategy

**MEDLINE**

1. Pregnancy/ or Pregnan*.tw.

2. Matern*.tw.

3. Gestation*.tw.

4. Preconcept*.tw.

5. Trying to conceive.tw.

6. 1 or 2 or 3 or 4 or 5

7. Midwifery/ or Midwi*.tw. or Nurse Midwives/

8. Obstetric*.tw. or Obstetrics/

9. Physicians, Family/ or Family Practice/ or General Practic*.tw. or General Practice/ or General Practitioners/

10. clinician*.tw.

11. ((health or healthcare) adj2 (profession* or work*)).mp.

12. Prenatal Care/ or (antenatal or prenatal).tw.

13. Preconception Care/

14. Perinatal Care/ or perinatal.tw.

15. Maternal Health Services/ or Maternity.tw. or Family Planning Services/

16. (family planning or fertility specialist*).tw.

17. 7 or 8 or 9 or 10 or 11 or 12 or 13 or 14 or 15 or 16

18. implement*.mp.

19. dissemin*.mp.

20. adopt*.mp.

21. practice*.mp.

22. organi?ational change*.mp.

23. diffus*.mp.

24. (system* adj2 change*).mp.

25. quality improvement*.mp.

26. transform*.mp.

27. translat*.mp.

28. transfer*.mp.

29. uptake*.mp.

30. sustainab*.mp.

31. institutionali*.mp.

32. routin*.mp.

33. maintenance.mp.

34. capacity.mp.

35. incorporat*.mp.

36. adher*.mp.

37. integrat*.mp.

38. scal*.mp.

39. ((polic* or guideline or practice* or program* or innovate*) adj5 (performance or feedback or audit* or monitor* or academic detailing or prompt* or reminder* or medical record* or record system* or incentive* or penalt* or mandat* or communicat* or social market* or professional development or network* or leadership* or opinion leader* or champion* or consensus* or change manage* or train* or educat* or resource* or material* or equipment or guideline)).mp.

40. 18 or 19 or 20 or 21 or 22 or 23 or 24 or 25 or 26 or 27 or 28 or 29 or 30 or 31 or 32 or 33 or 34 or 35 or 36 or 37 or 38 or 39

41. Fetal Alcohol Spectrum Disorders/

42. Alcohol Abstinence/ or Alcohol*.tw.

43. Drinking behaviour/ or Alcohol Drinking/ or Drinking/ or drink*.tw. or Binge Drinking/

44. Ethanol.tw. or Ethanol/

45. (Smok* adj2 (prevent* or reduc* or cessation or cease* or cigarette or tobacco)).mp.

46. Tobacco/ or Tobacco Smoking/ or Tobacco.tw.

47. Cigarette*.tw. or Tobacco Products/

48. Smoking Cessation/ or Nicotine Replacement.tw. or NRT.tw. or Smoking/

49. Weight gain/ or Weight gain.tw.

50. Nutri*.tw.

51. Diet.tw. or Diet/ or Healthy Diet/

52. (Food.tw. or Food/) and Nutrition/

53. Eat*.tw. or Eating/

54. Energy Intake.tw. or Energy Intake/

55. Physical Activit*.tw.

56. Exercise/ or Exercise.tw.

57. Sedentary Lifestyle/ or Physical Inactivit*.tw.

58. (Sedentary adj2 (Behavio* or Lifestyle)).mp.

59. Fitness.tw. or Physical Fitness/

60. 41 or 42 or 43 or 44 or 45 or 46 or 47 or 48 or 49 or 50 or 51 or 52 or 53 or 54 or 55 or 56 or 57 or 58 or 59

61. Randomized Controlled Trial/

62. Controlled Clinical Trial/

63. Clinical Trials as Topic/

64. Random Allocation/

65. Evaluation Studies/

66. Comparative Study/

67. random*.tw.

68. trial.tw.

69. groups.tw.

70. placebo.tw.

71. experiment*.tw.

72. (time adj series).tw.

73. (pretest or pre test or posttest or post test).tw.

74. impact.tw.

75. change*.tw.

76. evaluat*.tw.

77. effect*.tw.

78. 'before and after'.tw.

79. intervention*.tw.

80. program*.tw.

81. compare*.tw.

82. (control or controls* or controla* or controle* or controli or controll*).tw.

83. (Stepped wedge or staggered enrol*).tw.

84. 61 or 62 or 63 or 64 or 65 or 66 or 67 or 68 or 69 or 70 or 71 or 72 or 73 or 74 or 75 or 76 or 77 or 78 or 79 or 80 or 81 or 82 or 83

85. Ask*.tw.

86. Screen*.tw. or Mass Screening/

87. Assess*.tw. or Risk Assessment/

88. Advi?e.tw. or Health Education/

89. Assist*.tw.

90. Arrang*.tw.

91. (Refer*.tw. or Referral.mp.)

92. brief intervention.tw. or Motivational Interviewing/ or Psychotherapy, Brief/

93. 5A*.tw.

94. SBIRT.tw.

95. ((Care or practi?e*) adj (best or evidence* or recomm*)).tw.

96. 85 or 86 or 87 or 88 or 89 or 90 or 91 or 92 or 93 or 94 or 95

97. 6 and 17 and 40 and 60 and 84 and 96

**EMBASE**

1. Pregnancy/ or Pregnan*.tw.

2. Matern*.tw.

3. Gestation*.tw.

4. Preconcept*.tw.

5. Trying to conceive.tw.

6. 1 or 2 or 3 or 4 or 5

7. Midwife/ or Midwi*.tw. or Nurse Midwife/

8. Obstetric*.tw. or Obstetrics/

9. General Practic*.tw. or General Practice/ or General Practitioner/

10. clinician*.tw.

11. ((health or healthcare) adj2 (profession* or work*)).mp.

12. Prenatal Care/ or (antenatal or prenatal).tw.

13. Prepregnancy Care/

14. Perinatal Care/ or perinatal.tw.

15. Maternal Health Service/ or Maternity.tw. or Family Planning/

16. (family planning or fertility specialist*).tw.

17. 7 or 8 or 9 or 10 or 11 or 12 or 13 or 14 or 15 or 16

18. implement*.mp.

19. dissemin*.mp.

20. adopt*.mp.

21. practice*.mp.

22. organi?ational change*.mp.

23. diffus*.mp.

24. (system* adj2 change*).mp.

25. quality improvement*.mp.

26. transform*.mp.

27. translat*.mp.

28. transfer*.mp.

29. uptake*.mp.

30. sustainab*.mp.

31. institutionali*.mp.

32. routin*.mp.

33. maintenance.mp.

34. capacity.mp.

35. incorporat*.mp.

36. adher*.mp.

37. integrat*.mp.

38. scal*.mp.

39. ((polic* or guideline or practice* or program* or innovate*) adj5 (performance or feedback or audit* or monitor* or academic detailing or prompt* or reminder* or medical record* or record system* or incentive* or penalt* or mandat* or communicat* or social market* or professional development or network* or leadership* or opinion leader* or champion* or consensus* or change manage* or train* or educat* or resource* or material* or equipment or guideline)).mp.

40. 18 or 19 or 20 or 21 or 22 or 23 or 24 or 25 or 26 or 27 or 28 or 29 or 30 or 31 or 32 or 33 or 34 or 35 or 36 or 37 or 38 or 39

41. Fetal Alcohol Syndrome/

42. Alcohol Abstinence/ or Alcohol*.tw.

43. Drinking behaviour/ or Drinking/ or drink*.tw. or Binge Drinking/

44. Ethanol.tw.

45. (Smok* adj2 (prevent* or reduc* or cessation or cease* or cigarette or tobacco)).mp.

46. Tobacco/ or Tobacco.tw.

47. Cigarette*.tw.

48. Smoking Cessation/ or Nicotine Replacement.tw. or NRT.tw. or Smoking/

49. Body weight gain/ or Weight gain.tw.

50. Nutri*.tw.

51. Diet.tw. or Diet/ or Healthy Diet/

52. (Food.tw. or Food/) and Nutrition/

53. Eat*.tw. or Eating/

54. Energy Intake.tw. or Caloric Intake/

55. Physical Activit*.tw.

56. Exercise/ or Exercise.tw.

57. Sedentary Lifestyle/ or Physical Inactivit*.tw.

58. (Sedentary adj2 (Behavio* or Lifestyle)).mp.

59. Fitness.tw. or Fitness/

60. 41 or 42 or 43 or 44 or 45 or 46 or 47 or 48 or 49 or 50 or 51 or 52 or 53 or 54 or 55 or 56 or 57 or 58 or 59

61. Randomized Controlled Trial/

62. Controlled Clinical Trial/

63. Randomization/

64. Evaluation Study/

65. Comparative Study/

66. random*.tw.

67. trial.tw.

68. groups.tw.

69. placebo.tw.

70. experiment*.tw.

71. (time adj series).tw.

72. (pretest or pre test or posttest or post test).tw.

73. impact.tw.

74. change*.tw.

75. evaluat*.tw.

76. effect*.tw.

77. 'before.mp. and after'.tw.

78. intervention*.tw.

79. program*.tw.

80. compare*.tw.

81. (control or controls* or controla* or controle* or controli or controll*).tw.

82. (Stepped wedge or staggered enrol*).tw.

83. 61 or 62 or 63 or 64 or 65 or 66 or 67 or 68 or 69 or 70 or 71 or 72 or 73 or 74 or 75 or 76 or 77 or 78 or 79 or 80 or 81 or 82

84. Ask*.tw.

85. Screen*.tw. or Mass Screening/

86. Assess*.tw. or Risk Assessment/

87. Advi?e.tw. or Health Education/

88. Assist*.tw.

89. Arrang*.tw.

90. (Refer*.tw. or Referral.mp.)

91. brief intervention.tw. or Motivational Interviewing/ or Psychotherapy/

92. 5A*.tw.

93. SBIRT.tw.

94. ((Care or practi?e*) adj (best or evidence* or recomm*)).tw.

95. 85 or 86 or 87 or 88 or 89 or 90 or 91 or 92 or 93 or 94 or 95

96. 6 and 17 and 40 and 60 and 83 and 95

**MATERNITY AND INFANT CARE**

1. Pregnancy.de. or Pregnan*.tw.

2. Matern*.tw.

3. Gestation*.tw.

4. Preconcept*.tw.

5. Trying to conceive.tw.

6. 1 or 2 or 3 or 4 or 5

7. Midwifery.de. or Midwi*.tw. or Nurse-midwives.de.

8. Obstetric*.tw. or Obstetrics.de.

9. (Physicians, Family or Family Practice).de. or General Practic*.tw. or General Practice.de. or General Practitioners.de.

10. clinician*.tw.

11. ((health or healthcare) adj2 (profession* or work*)).mp.

12. Prenatal Care.de. or (antenatal or prenatal).tw.

13. Preconception Care.de.

14. Perinatal Care.de. or perinatal.tw.

15. Maternal Health Services.de. or Maternity.tw. or Family Planning Services.de.

16. (family planning or fertility specialist*).tw.

17. 7 or 8 or 9 or 10 or 11 or 12 or 13 or 14 or 15 or 16

18. implement*.mp.

19. dissemin*.mp.

20. adopt*.mp.

21. practice*.mp.

22. organi?ational change*.mp.

23. diffus*.mp.

24. (system* adj2 change*).mp.

25. quality improvement*.mp.

26. transform*.mp.

27. translat*.mp.

28. transfer*.mp.

29. uptake*.mp.

30. sustainab*.mp.

31. institutionali*.mp.

32. routin*.mp.

33. maintenance.mp.

34. capacity.mp.

35. incorporat*.mp.

36. adher*.mp.

37. integrat*.mp.

38. scal*.mp.

39. ((polic* or guideline or practice* or program* or innovate*) adj5 (performance or feedback or audit* or monitor* or academic detailing or prompt* or reminder* or medical record* or record system* or incentive* or penalt* or mandat* or communicat* or social market* or professional development or network* or leadership* or opinion leader* or champion* or consensus* or change manage* or train* or educat* or resource* or material* or equipment or guideline)).mp.

40. 18 or 19 or 20 or 21 or 22 or 23 or 24 or 25 or 26 or 27 or 28 or 29 or 30 or 31 or 32 or 33 or 34 or 35 or 36 or 37 or 38 or 39

41. Fetal Alcohol Spectrum Disorders.de.

42. Alcohol*.tw.

43. (Alcohol Drinking or Drinking).de. or drink*.tw.

44. Ethanol.tw. or Ethanol.de.

45. (Smok* adj2 (prevent* or reduc* or cessation or cease* or cigarette or tobacco)).mp.

46. Tobacco.de. or Tobacco.tw.

47. Cigarette*.tw.

48. Smoking Cessation.de. or Nicotine Replacement.tw. or NRT.tw. or Smoking.de.

49. Weight gain.de. or Weight gain.tw.

50. Nutri*.tw.

51. Diet.tw. or Diet.de.

52. (Food.tw. or Food.de.) and Nutrition.de.

53. Eat*.tw. or Eating.de.

54. Energy Intake.tw. or Energy Intake.de.

55. Physical Activit*.tw.

56. Exercise.de. or Exercise.tw.

57 Physical Inactivit*.tw.

58. (Sedentary adj2 (Behavio* or Lifestyle)).mp.

59. Fitness.tw. or Physical Fitness.de.

60. 41 or 42 or 43 or 44 or 45 or 46 or 47 or 48 or 49 or 50 or 51 or 52 or 53 or 54 or 55 or 56 or 57 or 58 or 59

61. Randomized Controlled Trials.de.

62. Controlled Clinical Trials.de.

63. Random Allocation.de.

64. Evaluation Studies.de.

65. Comparative Study.de.

66. random*.tw.

67. trial.tw.

68. groups.tw.

69. placebo.tw.

70. experiment*.tw.

71. (time adj series).tw.

72. (pretest or pre test or posttest or post test).tw.

73. impact.tw.

74. change*.tw.

75. evaluat*.tw.

76. effect*.tw.

77. 'before.mp. and after'.tw.

78. intervention*.tw.

79. program*.tw.

80. compare*.tw.

81. (control or controls* or controla* or controle* or controli or controll*).tw.

82. (Stepped wedge or staggered enrol*).tw.

83. 61 or 62 or 63 or 64 or 65 or 66 or 67 or 68 or 69 or 70 or 71 or 72 or 73 or 74 or 75 or 76 or 77 or 78 or 79 or 80 or 81 or 82

84. Ask*.tw.

85. Screen*.tw. or Mass Screening.de.

86. Assess*.tw. or Risk Assessment.de.

87. Advi?e.tw. or Health Education.de. or Antenatal Education.de.

88. Assist*.tw.

89. Arrang*.tw.

90. (Refer*.tw. or Referral.mp.)

91. brief intervention.tw. or Motivational Interviewing.tw. or Psychotherapy.de.

92. 5A*.tw.

93. SBIRT.tw.

94. ((Care or practi?e*) adj (best or evidence* or recomm*)).tw.

95. 84 or 85 or 86 or 87 or 88 or 89 or 90 or 91 or 92 or 93 or 94

96. 6 and 17 and 40 and 60 and 83 and 95

**CINAHL**

1. Pregnancy.mh

2. Matern$.tx.

3. Gestation$.tx.

4. Preconcept$.tx.

5. ‘Trying to conceive’.tx.

6. 1 or 2 or 3 or 4 or 5

7. Midwifery.mh

8. Obstetrics.mh

9. Physicians, Family.mh or Family Practice.mh

10. clinician$.tx.

11. ((health or healthcare) n2 (profession$ or work$)).ab

12. Prenatal Care.mh

13. Prepregnancy Care.mh

14. Perinatal Care.mh

15. Maternal Health Services.mh

16. (‘family planning’ or ‘fertility specialist$’).tx.

17. 7 or 8 or 9 or 10 or 11 or 12 or 13 or 14 or 15 or 16

18. implement$.tx.

19. dissemin$.tx.

20. adopt$.tx.

21. practice$.tx.

22. ‘organi?ational change$’.tx.

23. diffus$.tx.

24. (system$ n2 change$).tx.

25. ‘quality improvement$’.tx.

26. transform$.tx.

27. translat$.tx.

28. transfer$.tx.

29. uptake$.tx.

30. sustainab$.tx.

31. institutionali$.tx.

32. routin$.tx.

33. maintenance.tx.

34. capacity.tx.

35. incorporat$.tx.

36. adher$.tx.

37. integrat$.tx.

38. scal$.tx.

39. ((polic$ or guideline or practice$ or program$ or innovate$) n5 (performance or feedback or audit$ or monitor$ or ‘academic detailing’ or prompt$ or reminder$ or ‘medical record$’ or ‘record system$’ or incentive$ or penalt$ or mandat$ or communicat$ or ‘social market$’ or ‘professional development’ or network$ or leadership$ or ‘opinion leader$’ or champion$ or consensus$ or ‘change manage$’ or train$ or educat$ or resource$ or material$ or equipment or guideline)).tx.

40. 18 or 19 or 20 or 21 or 22 or 23 or 24 or 25 or 26 or 27 or 28 or 29 or 30 or 31 or 32 or 33 or 34 or 35 or 36 or 37 or 38 or 39

41. Fetal Alcohol Syndrome.mh

42. Alcohol$.tx.

43. Alcohol Drinking.mh

44. Ethanol.mh

45. (Smok$ n2 (prevent$ or reduc$ or cessation or cease$ or cigarette or tobacco)).tx.

46. Tobacco.mh

47. Tobacco Products.mh

48. Smoking Cessation.mh

49. Weight gain.mh

50. Nutri$.tx.

51. Diet.mh

52. Nutrition.mh

53. Eating.mh

54. Energy Intake.mh

55. ‘Physical Activit$’.tx.

56. Exercise.mh or Exercise.tx.

57. Life style, Sedentary.mh or ‘Physical Inactivit$.’tw.

58. (Sedentary n2 (Behavio$ or Lifestyle)).tx.

59. Physical Fitness.mh

60. 41 or 42 or 43 or 44 or 45 or 46 or 47 or 48 or 49 or 50 or 51 or 52 or 53 or 54 or 55 or 56 or 57 or 58 or 59

61. Randomized Controlled Trials.mh

62. Clinical Trials.mh

63. Random Sample.mh

64. Evaluation Research.mh

65. Comparative Studies.mh

66. random$.tx.

67. trial.tx.

68. groups.tx.

69. placebo.tx.

70. experiment$.tx.

71. (time n2 series).tx.

72. (pretest or ‘pre test’ or posttest or ‘post test’).tx.

73. impact.tx.

74. change$.tx.

75. evaluat$.tx.

76. effect$.tx.

77. 'before and after'.tx.

78. intervention$.tx.

79. program$.tx.

80. compare$.tx.

81. (control or controls$ or controla$ or controle$ or controli or controll$).tx.

82. (‘Stepped wedge’ or ‘staggered enrol$’).tx.

83. 61 or 62 or 63 or 64 or 65 or 66 or 67 or 68 or 69 or 70 or 71 or 72 or 73 or 74 or 75 or 76 or 77 or 78 or 79 or 80 or 81 or 82

84. Ask$.tx.

85. Screen$.tx. or

86. Assess$.tx. or

87. Advi$e.tx.

88. Assist$.tx.

89. Arrang$.tx.

90. Refer$.tx.

91. ‘brief intervention’.tx.

92. 5A$.tx.

93. SBIRT.tx.

94. ((Care or practi?e$) adj (best or evidence$ or recomm$)).tw.

95. 84 or 85 or 86 or 87 or 88 or 89 or 90 or 91 or 92 or 93 or 94

96. 6 and 17 and 40 and 60 and 83 and 95

**COCHRANE CENTRAL REGISTER OF CONTROLLED TRIALS**

"pregnancy" or Matern* or Gestation* or Preconcept* or “Trying to conceive”

AND

Midwifery or Obstetric or “General Practic*” or clinician* or “health professional” or “prenatal care” or “antenatal care” or “preconception care” or maternity or “family planning”

AND

implement or dissemin or adopt or practice or “organisational change*” or diffus* or “system change” or “quality improvement*” or transform* or translat* or transfer* or uptake* or sustainab* or institutionali* or routin* or maintenance or capacity or incorporat* or adher* or integrat* or scal* or polic* or guideline or practice* or program* or innovate* or performance or feedback or audit* or monitor* or “academic detailing” or prompt* or reminder* or “medical record*” or “record system*” or incentive* or penalt* or mandat* or communicat* or “social market*” or “professional development” or network* or leadership* or “opinion leader*” or champion* or consensus* or “change manage*” or train* or educat* or resource* or material* or equipment

AND

“Fetal Alcohol Spectrum Disorders” or alcohol or drinking or ethanol or tobacco or smok* or cigarette or “weight gain” or diet or nutri* or eating or “energy intake” or “Physical Activit*” or exercise or fitness

AND

Ask* or Screen* or Assess* or advise or advice or assist or arrang* or refer* or “brief intervention” or “motivational interviewing” or 5A* or SBIRT* or Care

**PROQUEST DISSERTATIONS AND THESES**

(Preconception OR prenatal) AND (midwife OR professional) AND (alcohol OR weight OR smoking) AND (implement OR adopt) AND care.

**WHO INTERNATIONAL CLINICAL TRIALS REGISTRY**

TITLE: preconception or prenatal or antenatal or pregnan* or profession* or midwi* or doctor*

CONDITION: alcohol or smoking or weight

INTERVENTION: adopt* or implement* or train* or resource* or educat* or guideline* or chang* or polic* or perform* or audit or feedback or prompt* or remind* or incentive or champion or program or quality improvement or mandat*
