# Supplementary material for: The effectiveness of implementation strategies in improving preconception and antenatal preventive care: a systematic review
Source: Implement Sci Commun. 2022 Nov 22;3:121. doi: 10.1186/s43058-022-00368-1 (PMC9682815; doi:10.1186/s43058-022-00368-1)
Supplement: Supplementary file 3 — Additional file 3. Characteristics of Included Studies. [file 43058_2022_368_MOESM3_ESM.docx]

**Supplementary File 3.** Characteristics of included studies

| **Study ID**  **Country**  **Trial design** | **Participants** | **Implementation strategies and Comparisons** | **Primary Outcomes – Effect of implementation strategies on improving the provision of preconception and antental care addressing modifiable risk factors** | | | **Secondary outcomes measured and reported** |
| --- | --- | --- | --- | --- | --- | --- |
|  |  |  | **Data Collection** | **Measures** | **Outcomes reported** |  |
| Aguilera et al. (2017)  **Country:** USA  **Study design:** Non-randomised controlled trial | **Setting**: antenatal care  **Services:** obstetrics practices (N=4)  **Health professionals:** doctors and nurses  **Allocation:** 2 practices to intervention and 2 to comparison condition | **Risk factor:** weight  **Implementation strategies:**   - educational meetings - educational materials   **Comparison:**   - usual practice/control   **Theoretical basis:** not reported  **Implementation measures:** acceptability | **Method:** health professional and women surveys  **Scale:** health professional: 5 point likert scale (never to always); women: 0-1 (no, yes)  **Sample for outcomes:**  **Health professional** N=22  IV: 11; C: 21  **Women** N=332  IV: 183; C: 149 | **Health professional**  **Advise:** discuss appropriate amount of weight gain during pregnancy; discuss risks of inadequate weight gain; discuss risks of excess weight gain  % (most of the time and always categories) C vs IV  **Women**  **Advise:** received advice about weight gain during pregnancy; discussion on the risks of gaining too much or not enough weight  % C vs IV | **Health professional** **Advise:** 73% vs 100%^b;^ 45% vs 54% ^b^; 45% vs 72% ^b^  **Women**  **Advise:** 66.4% vs 92.3%***; 51.5% vs 64.3%* | Secondary analysis of 308 medical records.  The proportion of women within weight recommendations (C vs IV)  36.9% vs 36.5%^a^ |
| Althabe et al. (2017)  **Country:** USA  **Study design:** Two-arm parallel cluster randomised trial | **Setting**: antenatal care  **Services:** antenatal clinics (N=20 clusters)  **Health professionals:** midwives and obstetrician/ gynaecologists  **Allocation:** 10 clusters allocated to intervention and 10 to comparison condition. | **Risk factor:** tobacco smoking  **Implementation strategies:**   - educational meetings - educational outreach visits, or academic detailing - local opinion leaders - reminders - tailored intervention   **Comparison:**   - educational meetings   **Theoretical basis:** Roger's Diffusion of Innovations Theory  **Implementation measures:** fidelity | **Method:** women surveys  **Scale:** 0-1 (no, yes)  **Sample for outcomes:** N=6828  IV: 3342 (pre: 1551; post: 1791); C: 3486 (pre: 1757; post: 1729) | **Ask:** asked about tobacco use (at > one visit)  **Advise:** advised about tobacco use (at > one visit)  **Assess:** assessed late quitters and continuous smokers are ready to quit (at > one visit)  **Assist:** assisted late quitters and continuous smokers in the quitting process (at > one visit)  **Arrange:** arranged follow-up with late quitters and smokers (at > one visit)  MRC C vs IV, AD (95% CI) | **Ask:** 3.9 vs 33.1, 29.2 (17.5; 38.0)**  **Advise:** 2.6 vs 29.0, 26.4 (13.9; 40.2)**  **Assess:** 5.3 vs 20.2, 14.9 (2.5; 32.7)*  **Assist:** 3.5 vs 25.0, 21.5 (10.6; 31.8)**  **Arrange:** 0.0 vs 2.7, 2.7 (0.0; 17.2)* | Sub-sample of women (IV: 863; C: 835) who quit or continued smoking during pregnancy and submitted cotinine analysis of saliva within 12 hours postpartum.  Quit smoking during pregnancy (C vs IV OR (95% CI), ROR (95% CI))  0.58 (0.43; 0.78) vs 0.74 (0.55; 1.00); 1.29 (0.84; 1.97)^a^ |
| Bakker et al. (2003)  **Country:** The Netherlands  **Study design:** RCT | **Setting:** antenatal care  **Services:** private midwifery practices (N=42)  **Health professionals:** midwives  **Allocation:** 22 clinics to intervention and 20 to comparison condition | **Risk factor:** tobacco smoking  **Implementation strategies:**   - clinical practice guidelines - educational meetings - educational materials   **Comparison:**   - usual practice/control   **Theoretical basis:** not reported  **Implementation measures:** fidelity, acceptability, feasability | **Method:** health professional and women surveys  **Scale:** health professional: 5 point likert scale (never to always); women: 0-1 (no, yes)  **Sample for outcomes:**  **Health professional**  N=69  IV: 37; C: 32  **Women:** N=95  IV: 44; C: 51 | **Health professional**  **Ask:** ask about smoking behaviour  **Advise:** advice to quit smoking; consequences of smoking during pregnancy  **Assess:** discuss barriers  **Assist:** set a quit date  **Arrange:** aftercare  M (SD) C vs I, B (SE)  **Women**  **Ask:** ask about smoking at intake  **Advise:** advice to quit  **Assess:** discuss barriers  **Assist:** set a quit date  M (SD) C vs IV, B (SE) | **Health professional**  **Ask:** 5.00 (0.00) vs 4.91 (0.37), -0.07 (0.07)^a^  **Advise:** 4.19 (1.03) vs 4.60 (0.77), 0.50 (0.24)*; 4.50 (0.95) vs 4.31 (0.99), -0.21 (0.26)^a^  **Assess:** 2.91 (1.15) vs 3.49 (1.12), 0.54 (0.30) ^a^  **Assist:** 1.63 (1.10) vs 3.63 (1.19), 1.69 (0.28)***  **Arrange:** 2.84 (0.99) vs 3.97 (0.89), 1.10 (0.25)***  **Women**  **Ask:** 0.72 (0.29) vs 0.91 (0.18), 0.17 (0.05)**  **Advise:** 0.64 (0.36) vs 0.85 (0.25), 0.22 (0.07)**  **Assess:** 0.11 (0.18) vs 0.38 (0.29), 0.25 (0.05)***  **Assist:** 0.03 (0.16) vs 0.33 (0.34), 0.22 (0.06)*** | Not reported |
| Bar-Zeev et al. (2019)  **Country:** Australia  **Study design:** Step-wedge cluster RCT | **Setting:** antenatal care  **Services:** Aboriginal Medical Services (N=6)  **Health professionals:** general practitioners, midwives, Aboriginal Health Workers and other allied health providers  **Allocation:** 6 services randomised into 3 clusters with the intervention delivered sequentially to each cluster | **Risk factor:** tobacco smoking  **Implementation strategies:**   - clinical practice guidelines - educational meetings - educational materials - tailored intervention   Comparison:   - usual practice/control   **Theoretical basis:** Behaviour Change Wheel and Theoretical Domains Framework  **Implementation measures:** fidelity, acceptability | **Method:** health professional suvey  **Scale:** 5 point likert scale (never to always)  **Sample for outcomes:**  pre=45; post=20  Matched pairs=15 | **Ask:** smoking status; nicotine dependence  **Advise:** brief advice to quit if smoking  **Assess:** discuss psychological context of smoking  **Assist:** provide cessation support to smokers;  recommend/prescribe NRT; refer to Quitline; refer to other specialist smoking cessation service  **Arrange:** follow-up within two weeks  % (often/always) pre vs post, OR (95% CI) | **Ask**: 98.7% vs 90.0%, 0.22 (0.01; 3.39)^a;^ 35.6% vs 60.0%, 3.09 (0.90; 10.65) ^a^  **Advise:** 63.6% vs 85.0%, 4.69 (0.92; 24.04) ^a^  **Assess:** 53.4% vs 45.0%, 0.72 (0.20; 2.61) ^a^  **Assist:** 48.9% vs 60.0%, 1.89 (0.49; 7.23) ^a^; 35.6% vs 30.0%, 0.91 (0.23; 3.62) ^a;^ 40.0% vs 40.0%, 1.06 (0.30; 3.73) ^a^; 35.5% vs 35.0%, 1.03 (0.29; 3.65) ^a^  **Arrange:** 31.1% vs 35.0%, 1.23 (0.33; 4.63) ^a^ | Not reported |
| Bazzo et al. (2015)  **Country:** Italy  **Study design:** Comparative study | **Setting:** antenatal care  **Services:** hospital obstetrics and gynecology units (N=4)  **Health professionals:** midwives  **Allocation:** 2 units to intervention and 2 to comparison condition | **Risk factor:** alcohol consumption  **Implementation strategies:**   - educational meetings   **Comparison:**   - usual practice/control   **Theoretical basis:** not reported  **Implementation measures:** not reported | **Method:** women survey  **Scale:** open ended, categorised into correct advice 0-1 (no, yes)  **Sample for outcomes:** N=67  IV:32; C: 35 | **Advise:** information on harmful effects on the fetus/newborn and/or completely abstain from alcohol during pregnancy  % C vs IV, RR (95% CI) | **Advise:** 20.0% vs 53.1%, 2.66 (1.27; 5.56)** | Not reported |
| Brownfoot et al. (2016)  **Country:** Australia  **Study design:** RCT | **Setting:** antenatal care  **Services:** antenatal clinics in a tertiary obstetrics hospital (N=1)  **Health professionals:** antenatal care providers  **Allocation:** 386 women randomised to the intervention and 396 to comparison condition | **Risk factor:** weight  **Implementation strategies:**   - educational materials - reminders   **Comparison:**   - usual practice   **Theoretical basis:** not reported  **Implementation measures:** not reported | **Method:** Medical record audit  **Scale:** no scale  **Sample for outcomes:** N=782  IV: 386; C: 396 | **Assess:** Number of times record of being weighed during pregnancy  M (SD), C vs IV | **Assess:** 1.8 (0.71) vs 5.6 (2.3)*** | 614 medical records had a weight recorded and were able to be audited for weight gain outcomes.  M (SD) weight gain per week, C vs IV  0.53 kg (0.24) vs 0.54 kg (0.28)^a^ |
| Campbell et al. (2006)  **Country:** Australia  **Study design:** RCT | **Setting:** antenatal care  **Services:** public hospitals (N=23; included in outcomes analyses N=22)  **Health professionals:** doctors and midwives  **Allocation:** 11 clinics to intervention and 11 to comparison condition | **Risk factor:** tobacco smoking  **Implementation strategies:**   - audit & feedback - educational materials - educational meetings - educational outreach visits, or academic detailing - tailored intervention   **Comparison:**   - educational materials   **Theoretical basis:** Roger's Diffusion of Innovations Theory  **Implementation measures:** fidelity | **Method:** women survey  **Scale:** 0-1 (no, yes)  **Sample for outcomes:** N=10994  IV: 6318 (pre: 3475; post: 2843)  C: 4676 (pre: 2374; post: 2302 (weighted N at post timepoints) | **Ask:** smoking status; discussed smoking at more than one visit  **Advise:** advice stop smoking completely; risk of smoking in pregnancy  **Assist:** methods could use to quit; discuss definite quit date  % (either midwife or doctor), C vs IV | **Ask:** 91.6% vs 92.9%^a;^ 12.4% vs 14.4%^a^  **Advise:** 38.7% vs 41.1%^a;^ 63.8% vs 64.5%^a^  **Assist:** 24.8% vs 30.0%^a^; 4.3% vs 5.8%^a^ | Smoking status was assessed for all women via self-report survey, corrected using expired air carbon monoxide (CO) data (>9 ppm indicated smoking).  % C vs IV  % quit (sub-sample of smokers who indicated smoking at first antenatal visit N=3121): 6.4% vs 10.5% ^a^  % current smokers (N=10954): 28.2% vs 24.8% ^a^ |
| Cooke et al. (2001)  **Country:** Australia  **Study design:** RCT | **Setting:** antenatal care **Services:** public hospital antenatal clinics (N=23)  **Health professionals:** doctors and midwives  **Allocation:** 11 clinics to intervention and 12 to comparison condition | **Risk factor:** tobacco smoking  **Implementation strategies:**   - audit & feedback - educational materials - educational meetings - educational outreach visits, or academic detailing - tailored intervention   **Comparison:**   - educational materials   **Theoretical basis:** Roger's Diffusion of Innovations theory  **Implementation measures:** penetration, adoption, sustainability | **Method:** health professional survey  **Scale:** 0-1 (no, yes)  **Sample for outcomes:** N=187  IV: 86; C: 101 | **Ask:** assessment of smoking  **Advise:** advice to quit; education about risk  **Assist:** methods to quit; encourage support person to assist, negotiate quit date; referral  **Arrange:** Follow-up discussion  % C vs IV, t-test | **Ask:** 97% vs 95%, 0.39^a^  **Advise**: 34% vs 46%, 2.92^a^; 92% vs 93%, 0.03^a^  **Assist:** 74% vs 86%, 3.92*; 47% vs 62%, 3.90*; 23% vs 47%, 10.94***; 55% vs 53%, 0.07^a^  **Arrange:** 68% vs 70%, 0.08^a^ | Not reported |
| Hajek et al. (2001)  **Country:** UK  **Study design:** RCT | **Setting:** antenatal care  **Services:** midwifery services in hospitals and community trusts (N=9)  **Health professionals:** midwives  **Allocation:** Of the 290 midwives who agreed to take part in the trial, 92 who were allocated to intervention and 86 allocated to the comparison condition ‘participated’ | **Risk factor:** tobacco smoking  **Implementation strategies:**   - educational meetings - educational materials - reminders   **Comparison:**   - usual practice/control   **Theoretical basis:** not reported  **Implementation measures:** feasibility | **Method:** women’s survey  **Scale:** 0-1 (no, yes)  **Sample for outcomes:** N=771  IV: 387 (unmotivated smokers: 100; motivated smokers: 287)  C*:* 384 (unmotivated smokers: 81; motivated smokers: 303) | **Ask:** discussed smoking; discussed smoking more than once  **Advise:** advised to set a date and stop abruptly; explained why smoking is dangerous  **Assist:** offered to find a buddy  % unmotivated smokers C vs IV; % motivated smokers C vs IV | **Ask:** 100% vs 100% ^a^; 98% vs 99%^a^; 38% vs 50%*; 40% vs 47% ^a^  **Advise:** 13% vs 29%*; 13% vs 64%***; 78% vs 94%**; 81% vs 95%***  **Assist:** 5% vs 42%***; 8% vs 68%*** | Smoking status assessed with 1120 women via survey and CO reading (with CO-reading<10 p.p.m indicating abstinence)  % C vs IV  Point prevalence abstinence at birth: 20% vs 22%^a^  Continuous abstinence (last 12 weeks of pregnancy) at birth: 17% vs 17%^a^  Continuous abstinence at 6 months post-birth: 8% vs 7%^a^ |
| Malta et al. (2016)  **Country:** Brazil  **Study design:** Non-randomised controlled trial | **Setting:** antenatal care  **Services:** primary care and family health units (N=17)  **Health professionals:** doctors and nurses  **Allocation:** 23 health professionals to intervention (one was excluded from outcome analyses) and 20 health professionals to comparison condition | **Risk factor:** weight (healthy eating and physical activity)  **Implementation strategies:**   - educational materials - educational meetings - local consensus process - tailored intervention   **Comparison:**   - usual practice/control   **Theoretical basis:** not reported  **Implementation measures:**  not reported | **Method:** women surveys  **Scale:** 0-1 (no, yes)  **Sample for outcomes:** N=281  IV: 140; C: 141 | **Advise:** Healthy eating; Leisure-time walking  % C vs I, PR (95% CI) | **Advise:** 19.1% vs 50.7%, 2.65 (1.82; 3.83)***; 33.3% vs 58.6%, 1.75 (1.34; 2.31)*** | Not reported |
| Manfredi et al. (2011)  **Country:** USA  **Study design:** Cluster RCT | **Setting:** antenatal care  **Services:** maternal and child health public health clinics (N=12; included in outcomes analyses N=8)  **Health professionals:** doctors and nurses  **Allocation:** 12 clinics allocated to 3 conditions. Only 2 of these conditions were relevant for the presented outcome analyses (4 clinics for intervention and 4 for comparison condition). | **Risk factor:** tobacco smoking  **Implementation strategies:**   - clinical practice guideline - educational materials - educational meetings - educational outreach visits, or academic detailing   **Comparison:**   - clinical practice guideline - educational materials - educational meetings   **Theoretical basis:** Roger's Diffusion of Innovations theory  **Implementation measures:** not reported | **Method:** women surveys  **Scale:** 0-1 (no, yes)  **Sample for outcomes:** N=854  IV: 371 (pre: 189; post: 182)  C: 483 (pre: 188; post: 295) | **Advise:** health professional advice  **Assist:** receipt of adjunct counselling  % (AOR) C vs IV | **Advise**: 54.2% (1.11) vs 69.8% (1.20)^a^  **Assist:** 9.5% (9.38) vs 17% (11.50)^a^ | Not reported |
| Mwansa-Kambafwile et al. (2011)  **Country:** South Africa  **Study design:** Controlled before and after study | **Setting:** preconception care  **Services:** public healthcare services (N=3 municipalities)  **Health professionals:** public sector healthcare workers  **Allocation:** 2 municipalities were allocated to intervention and 1 to comparison condition | **Risk factor:** alcohol consumption  **Implementation strategies:**   - educational materials - educational meetings   **Comparison:**   - usual practice/control   **Theoretical basis:** not reported  **Implementation measures:** not reported | **Method:** women survey  **Scale:** 0-1 (no, yes)  **Sample for outcomes:** N=375  IV: 284 (pre: 120; post: 164)  C: 91 (pre: 31; post: 60) | **Assess:** assess alcohol consumption  **Advise:** advised effects of maternal alcohol consumption on an unborn baby  % C vs IV, OR (95% CI)  (% not reported for assess outcome) | **Assess:** 1.15 (0.17; 1.03)^a^  **Advise:** 26.1% vs 87.9%, 5.07 (1.37; 6.96)* | Not reported |
| Omer et al. (2020)  **Country:** Ethopia  **Study design:** Cluster RCT | **Setting:** antenatal care  **Servcies:** antenatal units in health centres (N=20)  **Health professionals:** health officers, nurses and midwives  **Allocation:** matched pairs 10 to intervention and 10 to comparison condition | **Risk factor:** weight  **Implementation strategies:**   - educational materials - educational meetings - educatinal outreach visits, or academic detailing   **Comparison:**   - usual practice/control   **Theoretical basis:** not reported  **Implementation measures:** fidelity | **Method:** observations  **Scale:** 0-1 (no, yes)  **Sample for outcomes:** N=80  IV: 40; C: 40 | **Assess:** measured weight; monitored gestational weight gain  **Advise:** discussed possible options to practice recommendations; recommended achievable actions  % C vs IV, DID impact estimator (95% CI) | **Assess:** 2.50% vs 9.17%, 9.1 (0.47; 17.85)***; 0.00% vs 40.00%, 38.3 (26.43; 50.22)***  **Advise:** 4.20% vs 41.70%, 32.5 (19.99; 45.00)***; 38.30% vs 80.00%, 31.6 (11.79; 51.53)** | Not reported |
| Secker-walker et al. (1992)  **Country:** USA  **Study design:** RCT | **Setting:** antenatal care  **Services:** maternal infant care clinic (N=1)  **Health professionals:** obstetric and family practice residents  **Allocation:** 125 women who smoked at least one cigarette per day allocated to intervention and 125 to comparison condition | **Risk factor:** tobacco smoking  **Implementation strategies:**   - educational meetings - reminders   **Comparison:**   - educational meetings   **Theoretical basis:** not reported  **Implementation measures:** not reported | **Method:** women surveys  **Scale:** 0-1 (no, yes)  **Sample for outcomes:** N=250 women  IV: first visit: 125; second visit: 103  C: first visit: 125; second visit: 102 | **Ask:** talked about smoking first visit; talked about smoking second visit  **Advise:** advised to quit first visit; advised to quit second visit  **Assist:** set a quit date first visit; set a quit date second visit  % C vs IV | **Ask:** 95% vs 98%^a^; 66% vs 96%***  **Advise:** 92% vs 96%^a^; 52% vs 91%***  **Assist:** 14% vs 80%***; 7% vs 74%*** |  |
| Tsoh et al. (2010)  **Country:** USA  **Study design:** RCT | **Setting:** antenatal care  **Services:** community prenatal clinics (N=5)  **Health professionals:** community clinicians  **Allocation:** 23 pregnant smokers were allocated to intervention and 19 to comparison condition | **Risk factor:** tobacco smoking  **Implementation strategies:**   - reminders   **Comparison:**   - usual practice/control   **Theoretical basis:** not reported  **Implementation measures:** not reported | **Method:** women surveys  **Scale:** 0-1 (no, yes)  **Sample for outcomes:** N=42  IV: 23; C: 19 | **Advise:** advice at one or both visits  % C vs IV | **Advise:** 78.9% vs 95.7%** | Women self-reported smoking status at baseline and 2 month follow-up (N=42).  30 day abstinence  (% C vs IV) 15.4% vs 42.5%^a^  Mean decrease in number of days smoked: 1.1 vs 14.3**  Mean decrease in cigarettes smoked on a typical day: -0.1 vs 3.9^a^ |

*p<0.05, **p<0.01, ***p<0.001 ^a^ Not statistically significant at p<0.05 ^b^ Significance testing not conducted

AD Absolute Difference, RCT randomised controlled trial, IV intervention, C control, B baseline, F/U follow-up, CI Confidence Interval, MRC Median Rate Change, RD Relative Difference, ROR Relative Odds Ratio, RR Risk Ratio, M Mean, SD Standard Deviation, AOR Adjusted Odds Ratio
